# Morphospecies and Taxonomic Species Comparison for Hymenoptera

**DOI:** 10.1673/031.010.10801

**Published:** 2010-07-13

**Authors:** José G. B. Derraik, John W. Early, Gerard P. Closs, Katharine J. M. Dickinson

**Affiliations:** ^1^ Ecology, Conservation and Biodiversity Research Group, Department of Botany, University of Otago, PO Box 56, Dunedin, New Zealand; ^2^ Auckland Museum, Auckland Domain, Private Bag 92018, Auckland, New Zealand; ^3^ Ecology, Conservation and Biodiversity Research Group, Department of Zoology, University of Otago, PO Box 56, Dunedin, New Zealand; ^4^ Current address: Disease and Vector Research Group, Institute of Natural Sciences, Massey University, Private Bag 102904, North Shore MSC, Auckland, New Zealand

**Keywords:** parataxonomist, biodiversity, recognizable taxonomic units, Hymenoptera

## Abstract

The use of morphospecies as surrogates for taxonomic species has been proposed as an alternative to overcome the identification difficulties associated with many invertebrate studies, such as biodiversity surveys. Hymenoptera specimens were collected by beating and pitfall traps, and were separated into morphospecies by a non-specialist with no prior training, and later identified by an expert taxonomist. The number of Hymenoptera morphospecies and taxonomic species was 37 and 42, respectively, representing an underestimation error of 12%. Different families presented varying levels of difficulty, and although the species estimation provided by the use of morphospecies initially appeared to have a relatively minor error rate, this was actually an artefact. Splitting and lumping errors balanced each other out, wrongly suggesting that morphospecies were reasonable surrogates for taxonomic species in the Hymenoptera. The use of morphospecies should be adopted only for selected target groups, which have been assessed as reliable surrogates for taxonomic species beforehand, and some prior training to the non-specialist is likely to be of primary importance.

## Introduction

Human activities are causing a major decline in biodiversity, and it has been previously estimated that anthropogenic environmental change has accelerated extinction rates to 1000–10,000 times the natural rate (Lovejoy 1997). There is a daunting number of invertebrate species yet to be discovered, particularly in the tropics where the number of taxonomists is reduced and biodiversity funding is relatively scarce (Derraik et al. 2002). The use of ‘parataxonomists’ (Gamez 1991) or ‘biodiversity technicians’ (Cranston and Hillman 1992) has been consequently proposed to partly overcome this taxonomic impediment.

Parataxonomists could have a potentially important role in the implementation of rapid biodiversity assessments. Such assessments may be linked to the use of ‘recognizable taxonomic units’ (Cranston 1990; Trueman and Cranston 1997) or ‘morphospecies’ (Oliver and Beattie 1996a) rather than formally-described species. Morphospecies do not involve the identification of species *per se*, but rather the separation of taxa based on morphological characters that are easily observable (Derraik et al. 2002). The use of morphospecies as surrogates for taxonomic species in environmental monitoring and conservation studies appears to have been initially proposed by Kremen et al. (1993) and Oliver and Beattie (1996a). It was suggested that non-specialists could classify invertebrates to morphospecies without compromising scientific accuracy (Oliver and Beattie 1993, 1996a, 1996b; Beattie and Oliver 1994; Pik et al. 1999).

The issues surrounding the use of morphospecies for arthropod conservation have been previously discussed (Derraik et al. 2002; Krell 2004). This topic has generated considerable controversy, and it has lead to some heated debates in the past (Beattie and Oliver 1995, 1999; Brower 1995; Oliver and Beattie 1997; Goldstein 1997, 1999a, 1999b). Despite potential pitfalls, when applied with caution and in the right situations, morphospecies can be a useful technique for invertebrate studies, particularly where time and financial constraints exist, or in regions where detailed taxonomic information is limited.

In a previous assessment, accuracy of morphospecies separation was examined for Araneae, Coleoptera and Lepidoptera (Derraik et al. 2002). Several other groups were also collected during the same study project, and later identified by expert taxonomists (Derraik et al. 2001). Another speciose group in that investigation was the Hymenoptera (Derraik et al. 2001), one of the largest orders of insects, with 89 extant families and an estimated 300,000 species worldwide (Goulet and Huber 1993). The Hymenoptera has been suggested to be the most species-rich group in temperate regions (Gaston 1991), and it is therefore of interest from a biodiversity perspective. In this study, the accuracy of morphospecies separation for Hymenoptera in comparison to taxonomic species was compared.

## Methods and Materials

Invertebrate sampling was conducted in a modified native shrubland at 450 m elevation (45°30′S, 170°03′E) on the lower eastern slopes of the Rock and Pillar Range, South Island, New Zealand. For the collection of invertebrate specimens we focused on the two most important native shrub species in the shrubland community: *Olearia bullata* H. D. Wilson and Garn.-Jones (Asteraceae) and *Coprosma propinqua* A. Cunn. (Rubiaceae), two genera known to harbor a rich invertebrate fauna (Dugdale 1975; Patrick 2001). Thirty *O. bullata* and 30 *C. propinqua* shrubs were selected through random numbers and co-ordinates. They were sampled for invertebrates in late summer and early autumn (March and April 1999) using the beating method (Southwood 1978; Davis and Stork 1996; New 1998). Each plant received 10 downward strokes with a 1.5 m long metal rod, and the material that fell was collected on a polythene sheet (1.0 × 1.3 m) placed under the shrub. The material was sealed in a plastic bag, labelled, and frozen.

During the same period fifty pitfall traps (Davis and Stork 1996; New 1998) were set under 20 *O. bullata* and 20 *C. propinqua* plants. Ten other traps were scattered on nearby open patches of grassland dominated by the exotic *Agrostis capillaris* L. (browntop) and *Anthoxanthum odoratum* L. (sweet vernal). Each pitfall trap consisted of a PVC pipe 80 mm in diameter and 100 mm long, containing a plastic cup (opening 75 mm in diameter). Each cup was two-thirds filled with ethylene glycol, and a plastic lid supported 10–20 mm off the ground by bent wire covered the trap. The traps were emptied after two weeks.

An ecologist (JGBD), with no previous invertebrate taxonomic training, used a lowpower binocular microscope to conduct the initial sorting of invertebrates into morphospecies. No keys and only obvious external morphological features such as body shape and color patterns were used. No genitalia or other inconspicuous features were examined. The vials containing the numbered morphospecies were subsequently sent to a taxonomic expert to be identified as close to species level as possible. The accuracy of the morphospecies work was assessed as per Oliver and Beattie (1996a) and Derraik et al. (2002), using the formula below:

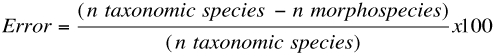


## Results and Discussion

Only adults were considered in this study, of which 178 hymenopteran specimens from 42 species were recorded. Fifteen specimens in the family Braconidae were excluded due to a lack of taxonomic expertise in New Zealand to properly identify them.

The number of Hymenoptera morphospecies and taxonomic species was 37 and 42, respectively. This represented an error of approximately 12%, thus true species richness was underestimated. As for the previous three orders looked at (Derraik et al. 2002), the numbers of taxonomic species and morphospecies identified were similar.

Table 1 provides an itemized description of the results by family. The Hymenoptera posed considerable difficulties for the untrained worker, but different families presented varying levels of difficulty. The Diapriidae in particular, was problematic, and the specimen separation led to a major underestimation of species richness in this group due to numerous lumping of separate taxonomic species into a single morphospecies (Table 1). In one instance, which occurred for Eulophidae as well, as many as four taxonomic species were incorrectly assigned to one morphospecies (Table 1).

The data in Table 1 demonstrate that, although the species estimation provided by the use of morphospecies initially appeared to produce a relatively low error rate (12%), this was an artefact of splitting and lumping errors balancing each other out. Overall, the non-specialist was only able to correctly assign specimens correctly to taxonomic species in 44% of the cases. As with the Coleoptera and Araneae examined previously (Derraik et al. 2002), similar levels of lumping and splitting errors led to a relatively close ratio of 37 morphospecies to 42 actual species (an underestimate of the number of taxonomic species of just under 12%).

The splitting mistake found in the Formicidae would most likely appear in other groups of social Hymenoptera (such as termites and bees). The morphological distinctions, including large size differences between the sexes and the various castes, would lead a non-specialist to identify them as different species, resulting in splitting mistakes. This problem however, would likely be easily corrected by pointing out to the parataxonomists beforehand some basic characters which allows species distinction for certain groups with relative ease.

**Table 1.**
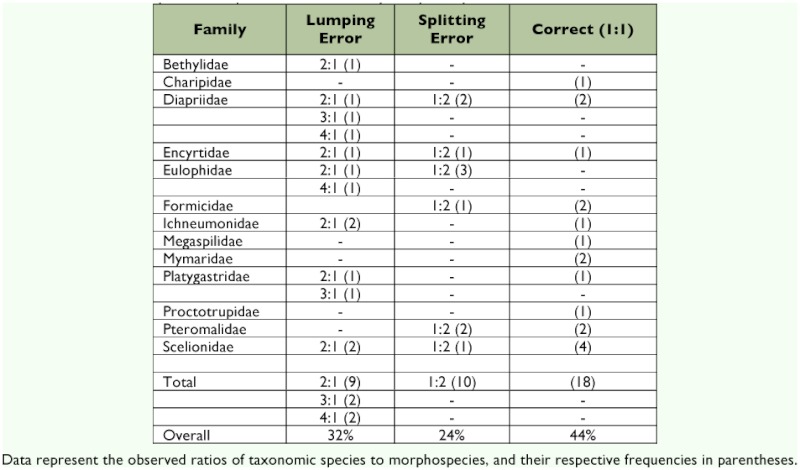
Outcome of specimens separation for each family of Hymenoptera.

However, some families can only be distinguished by careful examination of surface structure, sculpture and wing venation. This is particularly difficult for small microhymenoptera (1–3mm long) floating in a dish of alcohol. It is compounded by the often highly reflective exoskeleton, which requires critical lighting techniques for proper structural examination. Such is the case for Diapriidae and Eulophidae, which had the most inaccurate results of all families examined in this study (Table 1). These diverse and commonly occurring families are among those that created the most serious impediments for accurate morphospecies separation of Hymenoptera. Sexual dimorphism, particularly in antennal structure, is more readily observed and in this study there was a tendency to split taxonomic species based on this feature. Overriding this, individuals of the same sex of different species can appear more similar than males and females of the same species, and this led to lumping.

## Conclusions

Although when properly applied morphospecies can provide relatively quick and accurate estimates of species richness and turnover, this study and the previous results of Derraik et al. (2002) demonstrate that the accuracy of the final separation varies significantly between different arthropod orders. Despite the relatively small sample size in this study, it seems that, at least for the New Zealand fauna, some arthropod groups such as the smaller parasitic Hymenoptera present serious challenges for accurate identification even for expert taxonomists, which means that non-specialists are unlikely to do an accurate job. In the case of the Hymenoptera investigated here, one could speculate that if the sample size was larger, the accuracy of morphospecies separation would likely decrease. As a result, this study provides evidence that the use of morphospecies should be adopted only for selected target groups, where morphospecies have been assessed as reliable surrogates for taxonomic species beforehand.

It should be emphasized that the provision of prior training to the non-specialist is of primary importance, and would most likely improve the accuracy of the morphospecies separation (e.g. Barratt et al. 2003). Derraik et al. (2002) did not adequately emphasize the importance of some basic training prior to any parataxonomic work. Anecdotal evidence indicates that a couple of hours of instruction from an expert taxonomist on simple guidelines to separate potential species based solely on external morphology will greatly enhance morphospecies classification accuracy for certain groups (personal observation). We recommend therefore, that the extent of prior training and its effectiveness on morphospecies separation accuracy to be properly tested using control groups.
